# Characterization and clinical significance of right ventricular mechanics in pulmonary hypertension evaluated with cardiovascular magnetic resonance feature tracking

**DOI:** 10.1186/s12968-016-0258-x

**Published:** 2016-06-16

**Authors:** Maria Eduarda Menezes de Siqueira, Eduardo Pozo, Veronica R. Fernandes, Partho P. Sengupta, Karen Modesto, Sushilkumar Satish Gupta, Cayetana Barbeito-Caamaño, Jagat Narula, Valentin Fuster, Adriano Caixeta, Javier Sanz

**Affiliations:** The Zena and Michael A. Wiener Cardiovascular Institute/Marie-Josée and Henry R. Kravis Center for Cardiovascular Health, Icahn School of Medicine at Mount Sinai, New York, NY USA; Escola Paulista de Medicina, Universidade Federal de São Paulo, São Paulo, Brazil; Cardiology Department, Hospital Universitario de La Princesa, IIS-IP, Universidad Autónoma de Madrid, Madrid, Spain; Cardiology Department, Complexo Hospitalario Universitario A Coruña, Instituto de Investigación Biomédica de A Coruña, A Coruña, Spain; Mount Sinai Medical Center, One Gustave L Levy Place, Box 1030, New York, NY 10029 USA

**Keywords:** Pulmonary hypertension, Strain, Right ventricle

## Abstract

**Background:**

Prognosis in pulmonary hypertension (PH) is related to right ventricular (RV) function. Quantification of RV mechanics may offer additive value. The objective of our study is to determine the feasibility and clinical and prognostic value of RV strain analysis by cardiovascular magnetic resonance (CMR) based feature tracking (FT) in PH.

**Methods:**

We retrospectively enrolled 116 patients (age 52.2 ± 12 years, 73.6 % women) referred to CMR for PH evaluation who underwent right heart catheterization within 1 month. Using dedicated FT software, peak global longitudinal and circumferential RV strain and strain rates (GLS, GCS, GLSR, and GCSR, respectively) were quantified from standard cine images. Using multivariate regression analysis, we evaluated the associations of strain with a composite endpoint of death, lung transplantation, or functional class deterioration.

**Results:**

RV strain analysis was feasible in 110 (95 %) patients. Patients were classified into: Group A (no PH, normal right ventricular ejection fraction [RVEF]; *n* = 17), Group B (PH, normal RVEF; *n* = 26), or Group C (PH, abnormal RVEF; *n* = 67). All strain and strain rate values were reduced in Group C. Furthermore, GCSR was significantly reduced in Group B (-0.92 [-1.0/-0.7]; *p* < 0.001) compared to Group A (-1.12 [-1.3/-0.9]; *p* < 0.001). After adjustment for six clinically meaningful covariates, GLS (hazard ratio 1.06; *p* = 0.026), GLSR (hazard ratio 2.52; *p* = 0.04), and GCSR (hazard ratio 4.5; *p* = 0.01) were independently associated with the composite endpoint. GCSR successfully discriminated patients with and without events (*p* = 0.01).

**Conclusions:**

Quantification of RV strain with CMR-FT is feasible in the majority of patients, correlates with disease severity, and is independently associated with poor outcomes in PH.

**Electronic supplementary material:**

The online version of this article (doi:10.1186/s12968-016-0258-x) contains supplementary material, which is available to authorized users.

## Background

Pulmonary hypertension (PH) is a condition characterized by increased pressure and resistance in the pulmonary vasculature and is associated with high mortality. The prognosis of this disorder is directly related to right ventricular (RV) function, one of the main predictors of long-term outcome in patients with PH irrespective of its etiology [1–3]. Thus, accurate, reproducible, and clinically meaningful noninvasive methods for the detection and quantification of global or regional RV systolic function are important in the assessment of PH.

Echocardiography is the most widely available imaging modality for the evaluation of RV structure and function; however, image quality of the RV is often inadequate, and quantification can be subjective and limited by the complex geometry of this chamber [4]. Cardiovascular magnetic resonance (CMR) has emerged as the gold standard for the quantification of RV volume and ejection fraction (RVEF), particularly using steady-state free precession (SSFP) cine imaging [5]; however, other metrics of RV function such as quantification of myocardial deformation may allow detection of early abnormalities and provide independent prognostic information, as demonstrated in echocardiographic studies [6–8].

Recently, a novel method of “feature tracking” (FT) that allows quantification of myocardial deformation from CMR cine images without the need for additional imaging or lengthy analysis has been developed [9, 10]. Of note, CMR-FT has been validated against myocardial tagging for left ventricular (LV) strain analysis [11, 12] and recently against RV speckle tracking imaging for RV longitudinal strain evaluation in tetralogy of Fallot patients [13].

To the best of our knowledge, the significance of quantifying RV myocardial deformation in patients with PH using CMR-FT has not been investigated. Therefore, the aims of this study were to 1) evaluate the feasibility of performing routine RV strain analysis using CMR-FT, 2) characterize the pattern and severity of RV strain abnormalities in patients with PH, and 3) establish the prognostic significance of RV strain measurements in PH.

## Methods

### Patient population

We retrospectively evaluated patients referred for CMR evaluation of known or suspected chronic PH who also underwent right heart catheterization (RHC) within 1 month of the CMR. The presence of PH was defined as a mean pulmonary artery pressure >25 mm Hg at RHC [14]. Patients without PH and with RV dysfunction (RVEF <50 %) [15] suggestive of underlying myocardial disease, patients with cardiac shunts, or those with PH Groups 2-4 were excluded, resulting in 116 patients from Group 1 (pulmonary arterial hypertension) and Group 5 (unclear multifactorial mechanisms) of the PH Nice Classification [2]. After exclusion of 6 patients with inappropriate image quality due to arrhythmia, 110 patients were included in the present analysis. Of those, 17 patients who had no PH on RHC and a normal echocardiogram and CMR were used as controls.

Medical records were reviewed for clinical, hemodynamic, and CMR data. In addition, a composite endpoint of clinical worsening was recorded, defined as 1) all-cause mortality, 2) lung transplantation, or 3) worsening of New York Heart Association (NYHA) functional class. Only the most severe endpoint (death > transplant > worsening NYHA class) was used for analysis if more than one outcome occurred in the same patient. All deaths were confirmed by the Social Security Death Index. The Mount Sinai Institutional Review Board (New York, NY, USA) approved the study with a waiver of informed consent.

### CMR acquisition

CMR studies were performed on a 1.5 Tesla (MAGNETOM Sonata or MAGNETOM Avanto, Siemens Medical Solutions, Erlanger, Germany) or 3.0 Tesla (Ingenia Philips Healthcare, Best, The Netherlands) clinical magnets using 12- or 32-channel phased-array surface coils as receivers. Images were acquired during end-expiratory breath holds with retrospective electrocardiographic or pulse gating. Standard long-axis 4-chamber cine images were obtained using SSFP imaging. In addition, contiguous cine short-axis slices covering both ventricles from base to apex were also acquired with cine SSFP (typical acquisition parameters: repetition time/echo time 3.2–3.9/0.6–2 ms, flip angle 45–90°, slice thickness 6 mm, in-plane spatial resolution 1.5–2 mm, temporal resolution 33–45 ms, 25–30 reconstructed cardiac phases). RV and LV end-diastolic and end-systolic volumes and ejection fractions were obtained according to the Simpson method using specialized software (Argus, Siemens Medical Solutions or Extended MR WorkSpace, Philips Healthcare). To calculate RV mass, RV free wall epicardial and endocardial borders were traced on each end-diastolic short-axis. Volumes and mass were indexed to body surface area. Right atrial area was planimetered in the 4-chamber view in the phase showing maximal atrial dimension [16].

Late gadolinium enhancement (LGE) short and long axis images were obtained approximately 10 min after infusion of 0.2 mmol/kg of gadolinium-diethylenetriamine pentaacetic acid (Magnevist, Berlex Laboratories, Montville, New Jersey) using a T1-weighted, 2-dimensional, fast gradient echo sequence. The presence of LGE in the interventricular septum and/or RV insertion points was assessed visually as previously described [17]. No patient had LGE in other portions of the left ventricle.

### CMR-FT analysis

**Fig. 1:**
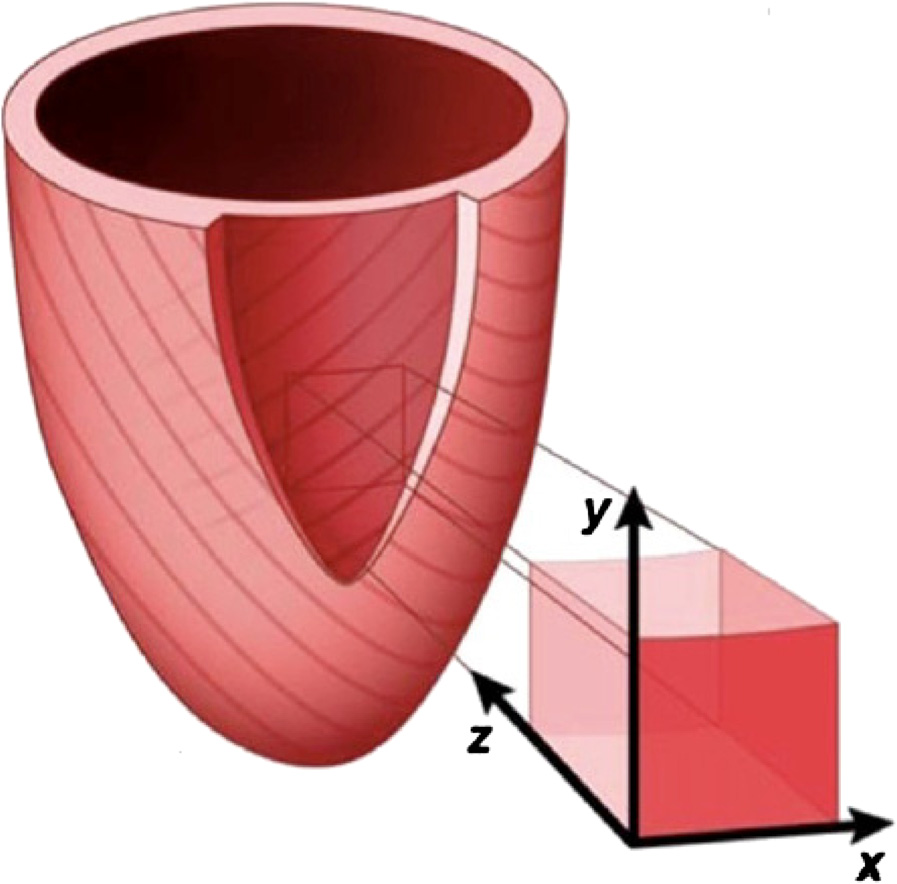
Schematic representation of myocardial fiber orientation and deformation in 3 orthogonal axis

Strain imaging measures the percentage change in myocardial deformation, while its derivative, strain rate, measures the rate of myocardium deformation over time (s^-1^) [18]. Myocardial fiber lengthening (expansion), by convention, is represented as a positive value for strain, while shortening (compression) is represented by a negative value. Myocardial fibers can deform in 3 spatial directions or planes (x, y, and z axis) (Fig. 1), respectively measured as circumferential, longitudinal, and radial strain.

**Fig. 2:**
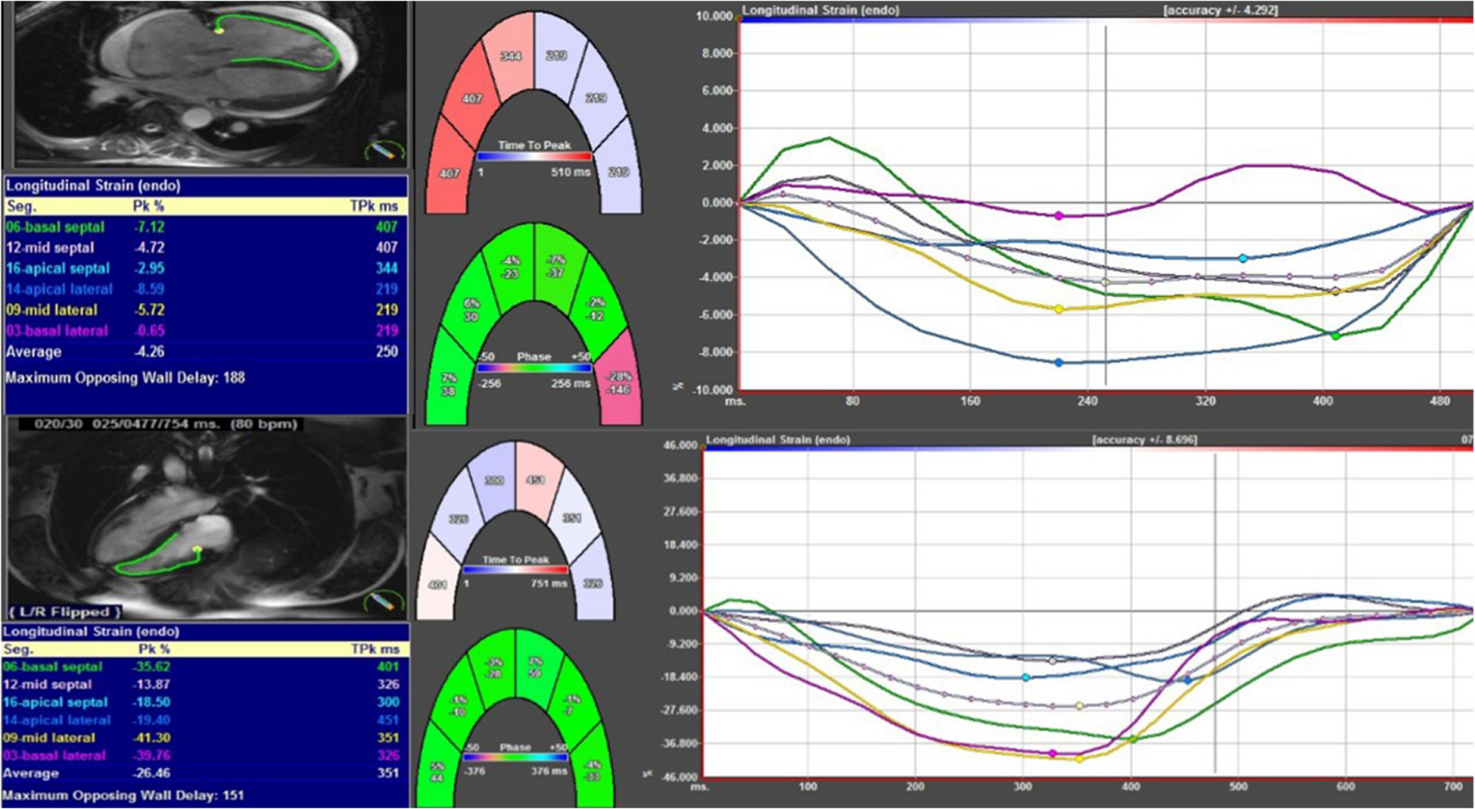
RV longitudinal strain analysis obtained from a four chamber view. Examples of longitudinal strain measurements in a patient without (*bottom*) and with pulmonary hypertension (*top*). The dotted line represents the average strain of the four segments

CMR-FT Diogenes software (2D CPA MR, version 1.1.2.36; TomTec Imaging Systems GmbH, Unterschleissheim, Germany) was used for deformation analysis on 3 short-axis (basal, mid, and apical levels) and a 4-chamber view. To ensure a standardized analysis for each patient, the basal slice in short axis view was defined as the first slice below the atrioventricular level showing circumferential LV myocardium, the mid-ventricular slice was localized at the level of both papillary muscles, and the apical slice at an apical location with the same distance to the mid-ventricular level as the basal slice. We used end diastolic phase to start the analysis. As described previously [19], RV endocardial contours were traced manually on one frame and then the software automatically propagated the contour throughout the remainder of the cardiac cycle. The contours were checked and manually adjusted if needed. RV global longitudinal strain (GLS) and strain rate (GLSR) were calculated as the average of peak systolic values from 2 basal, 2 mid, and 2 apical segments obtained from the 4-chamber view (Fig. 2). RV global circumferential strain (GCS) and strain rate (GCSR) were calculated as the average of 4 basal, 4 mid, and 4 apical segmental peak systolic values obtained from the short-axis views. In this study we decided not to evaluate radial deformation because it is our experience, as well as others’ [20], that this parameter is less reliably quantified using CMR-FT. The entire analytic process required approximately 4 min.

A single investigator (M.E.M.S) reviewed all CMR studies and performed the image analyses as detailed above. To assess intra-observer variability, the same investigator re-analyzed 20 studies 2 weeks after the first analysis. A second investigator (K.M) also evaluated 20 studies for the assessment of inter-observer reproducibility.

### RHC protocol

RHC was performed under fluoroscopic guidance using a Swan-Ganz catheter. Zero-pressure calibration was performed at the level of the mid-axillary line with the patient in the supine position. Baseline measurements included mean right atrial pressure, mean and systolic pulmonary artery pressures, pulmonary artery wedge pressure, cardiac index (obtained by thermodilution), pulmonary oxygen saturation, and pulmonary vascular resistance index. Systolic aortic pressure was measured by sphygmomanometry.

We classified patients according to the results of the RHC and CMR into one of three groups: A (control group; no PH and normal RVEF), B (PH and normal RVEF), or C (PH and decreased RVEF [<50 %]).

### Statistical analysis

Continuous variables were expressed as mean ± SD or median [interquartile range] depending on their distribution, and categorical variables were described as total number (percentage). When comparing patients with and without PH, the *χ*^2^ test and Fisher exact test were used for categorical variables where appropriate. Differences in normally and non-normally distributed continuous variables were established using a 2-tailed unpaired Student *t* test and Mann-Whitney *U* test, respectively, or in cases involving multiple groups, the ANOVA test and Kruskal Wallis test, respectively. Correlations between hemodynamic variables and those derived from CMR-FT strain were assessed by Pearson *r* or Spearman rho correlation coefficients, as appropriate. To test for intra- and inter-observer variability in strain measurements, we used Bland-Altman plots and intraclass correlation coefficient with a 2-way random model of absolute agreement.

CMR global strain parameters as well as other clinical, morphological, and hemodynamic variables significantly associated with the combined endpoint were identified. After discarding variables that showed collinearity, multivariate models were created using a selection of 6 clinically relevant variables in order to avoid overfitting. Then individual strain parameters were separately added into the models, and a Cox regression model was derived with a backward stepwise method for each strain/strain rate. Hereof, variables independently associated with the endpoint and predictive models were obtained. Results were presented as hazard ratios with 95 % confidence intervals. Receiver operating characteristic (ROC) curves were used to determine the accuracy of the global strain parameters in predicting the primary combined endpoint (death, transplant, or worsening of NYHA functional class). In addition, associations between the strain parameters and time to the primary endpoint were evaluated with adjusted survival Cox analysis using the best cut-off value derived from the ROC curves. Results were considered statistically significant when the 2-tailed *p* value was <0.05. Analyses were performed using SPSS 18.0 (IBM, Armonk, NY, USA).

## Results

### Patient characteristics

Demographic, clinical, hemodynamic, and CMR-derived parameters for the whole sample divided according to the presence or absence of PH and RV dysfunction are shown in Table 1. Among 110 patients, PH was absent in 17 (15.5 %) and present in 93 (84.5 %). There were 70 patients (75 %) with pulmonary arterial hypertension in PH Group 1 and 23 patients (25 %) in PH Group 5. The etiologic disease responsible for the placement in Group 1 was connective tissue disease in 25 patients, idiopathic PH in 23, portopulmonary syndrome in 11, human immunodeficiency virus infection in 10, and anorexigen abuse in 1. Among the PH Group 5 patients, sarcoidosis was the cause in 23 and sickle cell disease was the cause in 2. Diseases underlying the presumed diagnosis of PH in the 17 control subjects (Group A) included scleroderma in 5, sarcoidosis in 3, hepatitis in 2, and no disease in 7. Among those with PH, 26 patients had normal RVEF and 67 had decreased RVEF (comprising Groups B and C, respectively).

**Table 1:** Demographic, clinical, hemodynamic and cardiac magnetic resonance data according to the presence of pulmonary hypertension and right ventricular ejection fraction

Variable	Total (*n* = 110)	Group A (No PH and RVEF ≥50 %) (*n* = 17)	Group B (PH and RVEF ≥50 %) (*n* = 26)	Group C (PH and RVEF < 50 %) (*n* = 67)	*P*
Demographic data					
Age (yrs)	52.2 ± 12	52.6 ± 13	55 ± 12	51 ± 12	0.37
Female sex	81 (73.6 %)	15 (88.2 %)	22 (84.6 %)	44 (65.7 %)	0.06
Body surface area (m^2^)	1.79 ± 0.23	1.74 ± 0.17	1.76 ± 0.23	1.8 ± 0.24	0.369
Cardiovascular risk factors					
Diabetes	12 (10.9 %)	0	4 (15.4 %)	8 (11.9 %)	0.26
Hypertension	24 (21.8 %)	3 (17.6 %)	8 (30.8 %)	13 (19.4 %)	0.44
Hyperlipidemia	17 (15.5 %)	3 (17.6 %)	4 (15.4 %)	10 (14.9 %)	0.96
Smoking history	8 (7.3 %)	0	3 (11.5 %)	5 (7.5 %)	0.24
Medications					
Beta blockers	14 (12.7 %)	1 (5.8 %)	2 (8 %)	11 (18 %)	0.12
Calcium channel blockers	34 (30.9 %)	0	16 (61.5 %)	18 (26.9 %)	<0.001^** ŧ^
Diuretics	66 (60 %)	2 (13.3 %)	13 (52 %)	51 (83.6 %)	<0.001^** ŧ&^
Anticoagulants	38 (34.5 %)	2 (11.7 %)	8 (30.7 %)	28 (41.7 %)	0.053^&^
ERAs	31 (28.2 %)	0	4 (15.7 %)	27 (40.2 %)	<0.001^ŧ&^
Prostanoids	34 (30.9 %)	0	8 (30.7 %)	26 (38.8 %)	0.03^&^
PDIs	48 (43.6 %)	0	11 (42.3 %)	37 (55.2 %)	<0.001^**&^
Digoxin	38 (34.5 %)	2 (11.7 %)	4 (15.4 %)	32 (47.7 %)	0.001^ŧ &^
Corticosteroids	25 (22.7 %)	2 (11.7 %)	4 (15.4 %)	19 (28.3 %)	0.201
NYHA functional class ≥2	90 (81.8 %)	2 (11.7 %)	22 (84.6 %)	62 (92.5 %)	<0.001^**&^
Hemodynamic data					
Systolic aortic pressure (mm Hg)	122.1 ± 19	122.7 ± 20.3	132 ± 19.2	118.2 ± 18.1	0.009^ŧ^
PAWP (mm Hg)	9.8 ± 4.1	8.5 ± 2.8	11.5 ± 4.7	9.55 ± 4	0.05
Cardiac index (L/min/m^2^)	3.2 ± 1.1	3.71 ± 1	3.77 ± 1.5	2.91 ± 0.9	0.001^ŧ&^
Systolic PA pressure (mm Hg)	63.6 ± 24.7	29.6 ± 3.8	50.8 ± 13.1	72.1 ± 27.9	<0.001^&^
Mean PA pressure (mm Hg)	39.6 ± 14.9	17.1 ± 3.3	36.3 ± 10.6	46.6 ± 11.7	<0.001^**ŧ&^
RA pressure (mm Hg)	6 [23.1–35.6]	4 [3–6]	6 [4–10]	7 [5–14]	0.006^**&^
PVRI (Woods units × m^2^)	5.8 [2.7–9.3]	1.8 [1.2–2.4]	4 [2.1–8]	6.9 [5–10.4]	<0.001^**ŧ&^
PA oxygen saturation (%)	64.7 ± 10.97	72.6 ± 4.8	68.6 ± 6.9	61.1 ± 11.8	<0.001^ŧ&^
Cardiac magnetic resonance					
LVEF (%)	58.9 ± 9.8	63.3 ± 6	64.6 ± 5.2	55.5 ± 10.5	<0.001^ŧ&^
LVEDVi (mL/m^2^)	66.6 ± 17.4	69 ± 12.5	73 ± 25	63 ± 18.5	0.07
LVESVi (mL/m^2^)	27.6 ± 9.9	25.3 ± 6.6	26.1 ± 7	28.2 ± 11.3	0.30
RVEF (%)	42.15 ± 13.7	57.5 ± 4.6	55.2 ± 4.8	33.1 ± 9.4	<0.001^ŧ&^
RVEDVi (mL/m^2^)	98.8 [75.3–127]	73.1 [57.6–81.1]	85.4 [71.7–99]	116.4 [98–156]	<0.001^**ŧ&^
RVESVi (mL/m^2^)	56 [36.9–87.3]	28.7 [23.1–35.6]	39.6 [33.8–47.9]	78.6 [56.1–103]	<0.001^**ŧ&^
RV mass index (g/m^2^)*	25 [17.4–33]	15.5 [13.4–17.4]	18.8 [14.1–24.3]	30.2 [23.7–35.6]	<0.001**^ŧ^^&^
LGE	44 (40 %)	1 (5.9 %)	3 (11.5 %)	38 (56.7 %)	<0.001^ŧ&^
RA area (mm^2^)	24.54 ± 8.9	17.9 ± 3.3	21.9 ± 7	27.1 ± 9.5	0.001^ŧ&^
Clinical outcomes					
Death/transplant/worse NYHA class	78 (70.9 %)	4 (23.5 %)	12 (46.1 %)	62 (92.5 %)	<0.001^ŧ&^

Values are mean ± standard deviation, n (%), or median [interquartile range]

*ERA* endothelin receptor antagonist, *LGE* late gadolinium enhancement, *LVEF* left ventricular ejection fraction, *LVEDVi* left ventricular end-diastolic volume index, *LVESVi* left ventricular end-systolic volume index, *NYHA* New York Heart Association, *PA* pulmonary artery, *PAWP* pulmonary artery wedge pressure, *PDI* phosphodiesterase inhibitor, *PVRI* pulmonary vascular resistance index, *RA* right atrium, *RVEDVi* right ventricular end-diastolic volume index, *RVEF* right ventricular ejection fraction, *RVESVi* right ventricular end-systolic volume index

**Statistically significant differences between group A (control group) and group B

ŧ Statistically significant differences between group B and group C

& Statistically significant differences between group A and group C

As shown in Table 1, there were no differences among Groups A, B, and C with respect to age, sex, body surface area, or cardiovascular risk factors. Patients with PH were more likely to be symptomatic (NYHA functional class ≥2) and to use diuretics, phosphodiesterase inhibitors, and prostanoids. Those with preserved RVEF used calcium channel blockers more often, while those with RV dysfunction were more frequently treated with endothelin receptor antagonists and digoxin. As expected, mean pulmonary artery pressure and pulmonary vascular resistance index increased progressively from Group A to Group C. Patients with PH and RV dysfunction had lower cardiac index and pulmonary artery oxygen saturation, larger and more hypertrophic RV, larger right atrial dimensions, smaller RV and LV ejection fractions, and more frequent LGE.

### RV strain analysis

**Fig. 3:**
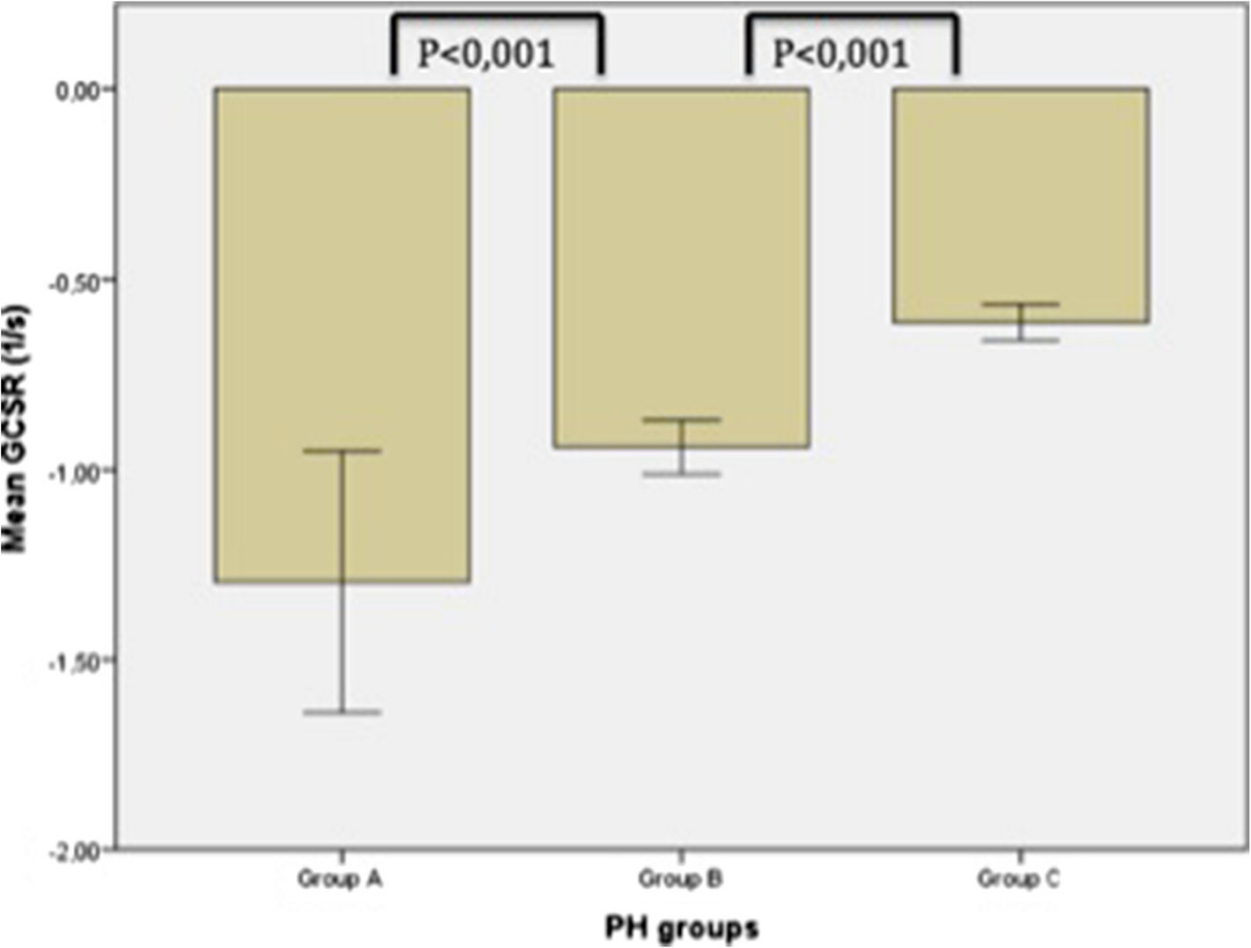
Right ventricular global circumferential strain rate (GCSR) values in the three groups. Among patients with preserved right ventricular ejection fraction, there was a significantly lower value for those with pulmonary hypertension (PH) (Group B) compared to those without PH (Group A)

Global RV strain and strain rate values for the whole sample and Groups A-C are presented in Table 2. All strain and strain rates were reduced in patients with PH and impaired RVEF in comparison with those without PH and those with preserved RVEF. In addition, GCSR was significantly reduced in the group with PH and preserved RVEF group compared to the control group (Table 2 and Fig. 3).

**Table 2:** Global right ventricular strain and strain rate

Strain Parameter	Total (*n* = 110)	Group A (No PH and RVEF ≥50 %) (*n* = 17)	Group B (PH and RVEF ≥50 %) (*n* = 26)	Group C (PH and RVEF < 50 %) (*n* = 67)	*P*
GLS (%)	-15.85 ± 6.4	-22.10 ± 4.7	-20.10 ± 5.3	-12.56 ± 4.8	<0.001^bc^
GCS (%)	-11.21 ± 4.9	-16.43 ± 4.7	-14.12 ± 3.5	-8.72 ± 3.5	<0.001^bc^
GLSR (s^-1^)	-1.09 ± 0.4	-1.46 ± 0.4	-1.33 ± 0.3	-0.89 ± 0.3	<0.001^bc^
GCSR (s^-1^)	-0.76 [-0.9 - (-0.5)]	-1.12 [-1.3 - (-0.9)]	-0.92 [-1.0 - (-0.7)]	-0.63 [-0.7 - (-0.4)]	<0.001^abc^

Values are mean ± standard deviation or median [interquartile range]

*GCS* global circumferential strain, *GCSR* global circumferential strain rate, *GLS* global longitudinal strain, *GLSR* global longitudinal strain rate, *PH* pulmonary hypertension, *RVEF* right ventricular ejection fraction

Continuous variables are expressed as mean ± standard

^a^Statistically significant differences between group A (control group) and group B

^b^statistically significant differences between group C and group B

^c^statistically significant differences between group A and group C

CMR-FT strain measurements demonstrated low inter- and intra-observer variability as indicated by the intraclass correlation coefficient (0.96/0.99; 0.94/0.98; 0.96/0.97; and 0.96/0.98 for GLS, GCS, GLSR, and GCSR, respectively; Additional file 1: Table S1 and Additional file 2: Figure S1).

### Correlations between RV deformation and disease severity

Correlations between RV strain/strain rates and hemodynamic and CMR measurements are shown in Additional file 3: Table S2. For these analyses, the absolute values (without the negative sign) of deformation indices were used. Overall, there were strong and moderate positive correlations of global strains and strain rates, respectively, with RVEF, and moderate negative corrrelations with RV mass index. There were also moderate negative correlations with RV volumes and mean pulmonary artery pressures, and weaker correlations with right atrial size and remaining hemodynamic parameters (except between GCS/GCSR and cardiac index). In addition, all deformation indices were reduced in patients with LGE (Additional file 4: Table S3).

### Global RV strain parameters and clinical outcome

Median duration of follow-up was 730 days (range 190 to 1585 days). There were 78 events: 38 deaths (34.5 %), 2 lung transplantations (1.8 %), and 38 cases of NYHA class worsening (34.5 %). Patients with PH and RVEF <50 % had a greater incidence of events (*p* < 0.001) compared to those with either PH and normal RVEF or no PH. There was no statistically significant difference regarding the composite endpoint between Groups A and B (Table 1).

The associations of all invasive and non-invasive variables with the combined endpoint are reported in Table 3. In brief, the medication use and functional class differed between those who did or did not have events. Among RHC parameters, systemic and pulmonary pressures, cardiac index, pulmonary vascular resistance index, and pulmonary oxygen saturation were associated with the primary endpoint. Similarly, among CMR measurements, reduced biventricular ejection fractions and LV end-diastolic volume, enlarged RV and right atrium, RV hypertrophy and presence of LGE each were more common in patients experiencing events. All strain and strain rates were also decreased in the event-positive group.

**Table 3:** Univariate analysis of the associations with the combined endpoint

Variables	Death/Transplant/Worse NYHA (*n* = 78)	No endpoint (*n* = 32)	*P*
Clinical, CMR and hemodinamics variables			
Age (yrs)	53 ± 12	50 ± 12	0.35
Sex (female)	54 (69.2 %)	27 (84.4 %)	0.15
Body surface area (m^2^)	1.8 ± 0.25	1.7 ± 0.22	0.45
Use of medication	71 (91 %)	10 (31 %)	<0.001
Smoking	5 (6.4 %)	3 (9.4 %)	0.059
NYHA class ≥2	71 (91 %)	3 (9.3 %)	<0.001
Systolic aortic pressure (mm Hg)	119 ± 18	127 ± 20.5	0.03
PAWP (mm Hg)	9.7 ± 4.2	10.1 ± 4.7	0.38
Cardiac index (L/min/m^2^)	3 ± 1	3.6 ± 1.57	0.04
Systolic PA pressure (mm Hg)	73.2 ± 20.3	58.1 ± 19.1	0.004
Mean PA pressure (mm Hg)	44.6 ± 11.8	27.5 ± 12.2	<0.001
RA pressure	9.4 ± 7.9	6.4 ± 3.0	0.18
PVRI (Woods units x m^2^)	6.6 [3.9–10.3]	2.4 [1.5–6.8]	0.004
PA sat (%)	62 ± 11.6	71 ± 4.8	0.002
LVEF (%)	56.9 ± 10.8	62.5 ± 5.5	<0.001
LVEDVi (mL/m^2^)	64 ± 18	72 ± 14	0.036
LVESVi (mL/m^2^)	27.7 ± 10.9	27.3 ± 6.7	0.82
RVEF (%)	37.38 ± 12.4	53.7 ± 9.4	<0.001
RVEDVi (mL/m^2^)	112 [87.6–144.6]	75 [69.9–97.9]	0.01
RVESVi (mL/m^2^)	70 [44.9–95.4]	35.2 [28.7–48.3]	<0.001
RV mass index (gr/m^2^)	29.8 [23.5–35.1]	18.4 [14.3–22.9]	0.001
LGE	39 (50 %)	5 (15.6 %)	0.01
RA area	26 ± 9.4	20 ± 5.8	0.001
Strain parameters			
GLS (%)	- 13.57 ± 5.5	-19.01 ± 5.7	<0.001
GCS (%)	-9.6 ± 4.2	-12.58 ± 3.7	0.007
GLSR (s^-1^)	-0.94 ± 0.3	-1.33 ± 0.4	<0.001
GCSR (s^-1^)	-0.67 [-0.7 - (-0.4)]	-0.92 [-1.1 - (-0.8)]	<0.001

Values are mean ± standard deviation, n (%), or median [interquartile range]

*GCS* global circumferential strain, *GCSR* global circumferential strain rate, *GLS* global longitudinal strain, *GLSR* global longitudinal strain rate, *LGE* late gadolinium enhancement, *LVEF* left ventricular ejection fraction, *LVEDVi* left ventricular end-diastolic volume index, *LVESVi* left ventricular end-systolic volume index, *NYHA* New York Heart Association, *PA* pulmonary artery, *PAWP* pulmonary artery wedge pressure, *PVRI* pulmonary vascular resistance index, *RA* right atrium, *RVEDVi* right ventricular end-diastolic volume index, *RVEF* right ventricular ejection fraction, *RVESVi* right ventricular end-systolic volume index

**Fig. 4:**
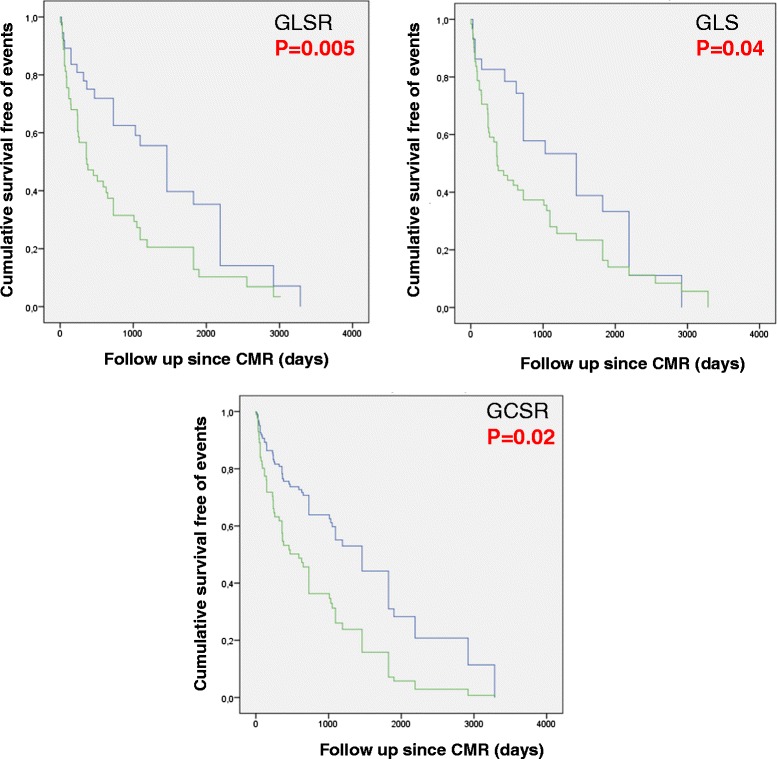
Kaplan Meyer survival curves for survival free of the combined endpoint according to CMR global RV strain parameters

**Fig. 5:**
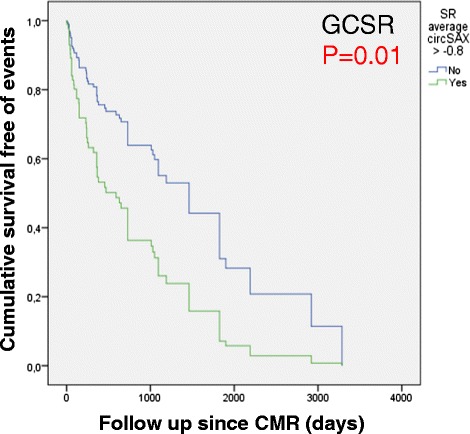
Cox survival curve for survival free of the combined endpoint according to CMR global RV strain parameter cutoff point. GCSR cut off point > -0.8 s^-1^ was able to predict events in the multivariate model

From non–strain-related variables associated with outcomes (Table 3) the following were selected for inclusion in the Cox analysis based on their univariate associations and known clinical value: NYHA class ≥2, cardiac index, pulmonary artery oxygen saturation, mean pulmonary artery pressure, RV mass index, and RVEF. We did not include RV end-diastolic volume index, systolic pulmonary pressure, or pulmonary vascular resistance for multivariate analysis since they showed collinearity with previous variables, and to avoid model overfitting. Table 4 shows the final predictive models with the corresponding adjusted hazard ratios obtained when each global strain parameter was introduced into the model. When the strain parameters were analyzed as continuous variables, GLS (together with cardiac index, RV mass index and mean pulmonary artery pressure), GLSR (together with cardiac index and RV mass index), and GCSR (together with cardiac index, RV mass index and mean pulmonary artery pressure) were each independently associated with the combined endpoint, whereas GCS was not. GLS, GLSR, GCR and GCSR cut-off points, obtained from optimal values in the ROC curves (GLS -17 %, GLSR -1.1 s^-1^, GCS -12 %, and GCSR -0.8 s^-1^) were able to differentiate patients that presented the combined end-point during the follow-up, as shown in Kaplan-Meyers curves (Fig. 4). However, only GCSR cut-off point remained as independent outcome predictor after adjusting in the multivariate model (Fig. 5).

**Table 4:** Cox proportional adjusted hazard ratio for final multivariate models of each RV strain parameter

Models	Hazard ratio	95 % Confidence Interval	*P*
GLS	1.06	1–1.12	0.026
Cardiac index	0.59	0.4–0.79	0.030
RV mass index	1.02	1–1.04	0.006
Mean PA pressure	0.97	0.95–0.99	0.02
ᅟ			
RVEF	0.97	0.94–0.99	0.03
Cardiac index	0.66	0.49–0.90	0.009
RV mass index	1.02	1–1.04	0.02
Mean PA pressure^a^	0.97	0.95–0.99	0.02
ᅟ			
GLSR	2.52	1.03–6.1	0.04
Cardiac index	0.65	0.48–0.89	0.008
RV mass index	1.03	1.01–1.05	0.033
ᅟ			
GCSR	4.5	1.3–15.6	0.01
Cardiac index	0.61	0.46–0.81	0.001
RV mass index	1.02	1–1.04	0.006
Mean PA pressure	0.97	0.95–0.99	0.01

^a^Model including global circumferential strain, not shown

*GCSR* global circumferential strain rate, *GLS* global longitudinal strain, *GLSR* global longitudinal strain rate, *PA* pulmonary artery, *RVEF* right ventricular ejection fraction

An additional multivariate analysis was also performed including guideline-recommended variables for prognostication in PH [21]: NYHA class ≥2, cardiac index, pulmonary artery oxygen saturation, right atrial pressure and RVEF. GLS (together with cardiac index), GLSR (together with RVEF), and GCSR (together with cardiac index) were each independently associated with the combined endpoint, and again GCS was not (Additional file 5: Table S4). Finally, when all strain parameters were forced together into the first model, the final variables retained were GCSR, cardiac index, mean pulmonary artery pressure, and RV mass index (Additional file 6: Table S5).

## Discussion

The major findings of our study are as follows: 1) Systematic evaluation of RV strain in PH with CMR-FT is feasible and reproducible, 2) measures of RV strain correlate with disease severity, 3) reduced RV strain indices are associated with subsequent clinical deterioration, and 4) GCSR specifically is associated with outcomes even after adjustment for other prognostic predictors in PH.

RV function is the main predictor of survival in PH patients [1–3]. While echocardiography remains the most commonly used modality to assess this factor in clinical practice, CMR is today considered the gold standard for the quantification of RV volumes and ejection fraction [5]; however, other measures of myocardial deformation may be of interest and provide additive information regarding RV performance. Myocardial tissue tagging with CMR is considered the noninvasive reference method to assess LV regional deformation [22, 23]. Although feasible [24], its application to the right chamber is limited mostly because of the relatively thin chamber wall, and the method requires laborious offline post-processing analysis. In contrast to conventional tagging, strain-encoded CMR is an alternative technique that provides direct myocardial strain imaging, and its capability of quantifying RV strain in PH has been reported [25, 26]. However, the sequence may not be widely available, and additional scanning is required. Therefore, a method like FT that can be applied in routinely acquired cine images can be potentially more practical and time saving.

CMR-FT of the RV has been successfully used to demonstrate RV dysfunction in congenital heart disease [13, 27] and arrhythmogenic RV dysplasia [28] with good inter-study and inter-observer reproducibility for RV global longitudinal strain measures. More recently, Ohyama et al. [19] quantified RV longitudinal strain from cine images in 26 patients with PH using a different pixel-based multimodality tissue tracking method. They validated it against strain encoded imaging and also reported high reproducibility, although they could not show differences with normal volunteers. In our much larger series of 116 patients, strain could not be evaluated only in 6 patients (5 %). In addition, we were able to demonstrate significant reductions in GLS, GCS, GLSR, and GCSR compared with individuals lacking PH. Similar to Ohyama and colleagues, reductions in strain parameters correlated with disease severity whether measured as impairment in hemodynamics or abnormalities in right heart chambers. Importantly, decreased myocardial deformation was already noted in the presence of PH alongside preserved RVEF, although this reduction reached statistical significance in comparison with individuals without PH only for GCSR, perhaps due to sample size limitations. This finding indicates a potential role for this parameter for noninvasive detection of PH or of early RV decompensation.

To our knowledge, this is the first study to show the relationship between CMR-FT-derived strain analysis and outcomes in patients with PH. After adjustment for 6 covariates with known clinical and prognostic value (namely functional class, cardiac index, pulmonary artery oxygen saturation, mean pulmonary artery pressure, RV mass index, and RVEF), GLS, GLSR, and GCSR remained independently associated with the combined endpoint of death, lung transplantation, or decrease in NYHA class. Our findings are consistent with those reported in the echo literature using speckle-tracking based analysis of RV deformation. Haeck et al evaluated 150 patients with PH and demonstrated that RV GLS is a significant tripled determinant of all-cause mortality risk compared to patients with higher GLS (threshold of < −19 %) [7]. Sachdev et al also found that patients with PH and lower RV strain and strain rate values had poorer survival [29]. In a prospective study with a large cohort of 575 patients, RV strain predicted outcome independent of other clinical and echocardiographic variables [8]. Interestingly, Hardegree et al performed a study to assess whether serial quantitative assessment of RV strain by speckle-tracking was affected by PH-specific treatments and concluded that strain imaging independently predicts clinical deterioration and mortality in PH patients after the institution of medical therapy [30]. Although GLS and GLSR were also predictors of events in our study, probably reflecting the larger dependence of the RV on longitudinal shortening [31], survival Cox analysis demonstrated GCSR to be superior in identifying patients at heightened risk of events. Strain rate describes the degree of change in myocardial deformation with respect to time and has been found to reflect myocardial contractility better than strain parameters, which are more pre-load and after-load dependent and may change with ventricular dimensions [32, 33]. Normal RV function is highly dependent on longitudinal shortening [34]; however, it has been suggested that transverse wall motion may be a better reflection of systolic function in PH than longitudinal motion, and it is possible that as PH advances and the RV becomes more hypertrophic, circumferential deformation becomes relatively more important resembling LV contractility patterns [35]. In addition, we have previously demonstrated heterogeneity of regional RV function in PH even before RVEF decreases [36]. Thus, evaluating RV myocardial deformation may identify subclinical RV dysfunction before the development of abnormalities on conventional measures of RV performance. Further evidence that strain reflects RV performance beyond RVEF (similar to findings in the LV) is the independent value of strain when adjusting for RVEF shown in this study.

### Limitations

This was a single-center, retrospective study performed at a large tertiary hospital, so the inherent limitations of this study design cannot be avoided, specifically the possibilities of referral bias and residual confounding. We tried to limit sample heterogeneity by including only patients in PH Group 1 and Group 5 but etiologies of PH and underlying conditions still varied, thus we cannot exclude the possibility that other diseases influenced RV strain. However, this reflects the clinical reality of a relatively rare disorder. Although the number of events was rather large at 78, we were unable to include every single variable associated with outcomes in the multivariate models. Nonetheless, we could find significant associations between RV strain parameters and events risk after adjusting for 6 clinically and prognostically relevant covariates. The control group was not free of disease, a fact that could interfere with the strain values. Nevertheless, biventricular volumes and ejection fractions were normal, and no LGE was noted in these patients except one focal case at the RV insertion point in a patient with scleroderma. CMR and RHC were not performed simultaneously, which may reduce the associations between hemodynamic parameters and RV deformation; however, the interval between the 2 exams was not more than 1 month. The temporal resolution of the CMR cine images (33–45 ms) was lower than commonly recommended for strain rate evaluation; therefore, true strain rate values are likely underestimated. Finally, the CMR-FT strain analysis software used in this study was designed primarily for LV strain analysis and adapted for use on the RV; strain analysis software specific to RV has yet to be developed.

## Conclusions

CMR-FT assessment of RV strain can be successfully incorporated into a comprehensive CMR exam protocol for PH and may represent a valuable noninvasive method of evaluation that could be widely adopted at PH centers. Routine RV strain measurement using CMR-FT is feasible in PH patients, correlates with disease severity, and is associated with clinically relevant outcomes.

## Abbreviations

CMR, cardiovascular magnetic resonance; FT, feature-tracking; GCS, global circumferential strain; GCSR, global circumferential strain rate; GLS, global longitudinal strain; GLSR, global longitudinal strain rate; LGE, late gadolinium enhancement; LV, left ventricular; NYHA, New York Heart Association; PH, pulmonary hypertension; RHC, right heart catheterization; RV, right ventricular; RVEF, right ventricular ejection fraction; SSFP, steady-state free precession

## Additional files

Additional file 1: Table S1. Inter- and intra-observer agreement. (DOCX 48 kb) Additional file 2: Figure S1. Bland-Altman plots for inter-observer agreement. (PPTX 509 kb) Additional file 3: Table S2. Correlation coefficients of right ventricular strain indices (absolute values) with CMR and RHC-derived parameters. (DOCX 82 kb) Additional file 4: Table S3. Strain parameters in patients with and without late gadolinium enhancement. (DOCX 47 kb) Additional file 5: Table S4. Cox proportional adjusted hazard ratio for final multivariate alternative model of each RV strain parameter. (DOCX 45 kb) Additional file 6: Table S5. Cox proportional adjusted hazard ratio for final multivariate model including all RV strain parameters. (DOCX 39 kb)
