# Supplementary material for: Characterization and clinical significance of right ventricular mechanics in pulmonary hypertension evaluated with cardiovascular magnetic resonance feature tracking
Source: J Cardiovasc Magn Reson. 2016 Jun 16;18:39. doi: 10.1186/s12968-016-0258-x (PMC4910232; doi:10.1186/s12968-016-0258-x)
Supplement: Additional file 1: Table S1. — Inter- and intra-observer agreement. (DOCX 48 kb) [file 12968_2016_258_MOESM1_ESM.docx]

**Supplemental Table 1. Inter- and intra-observer agreement**

|  | Inter-observer variability Intraclass correlation (95% CI) | Intra-observer variability Intraclass correlation (95% CI) |
| --- | --- | --- |
| GLS | 0.96 (0.85-0.99) | 0.99 (0.97-0.99) |
| GCS | 0.94 (0.77-0.98) | 0.98 (0.93-0.99) |
| GLSR | 0.96 (0.85-0.99) | 0.97 (0.91-0.99) |
| GCSR | 0.96 (0.86-0.99) | 0.98 (0.94-0.99) |

CI = confidence interval; GCS = global circumferential strain; GCSR = global circumferential strain rate; GLS = global longitudinal strain; GLSR = global longitudinal strain rate.
