# Supplementary figures and images for: Characterization and clinical significance of right ventricular mechanics in pulmonary hypertension evaluated with cardiovascular magnetic resonance feature tracking
Source: J Cardiovasc Magn Reson. 2016 Jun 16;18:39. doi: 10.1186/s12968-016-0258-x (PMC4910232; doi:10.1186/s12968-016-0258-x)

## Slide 1
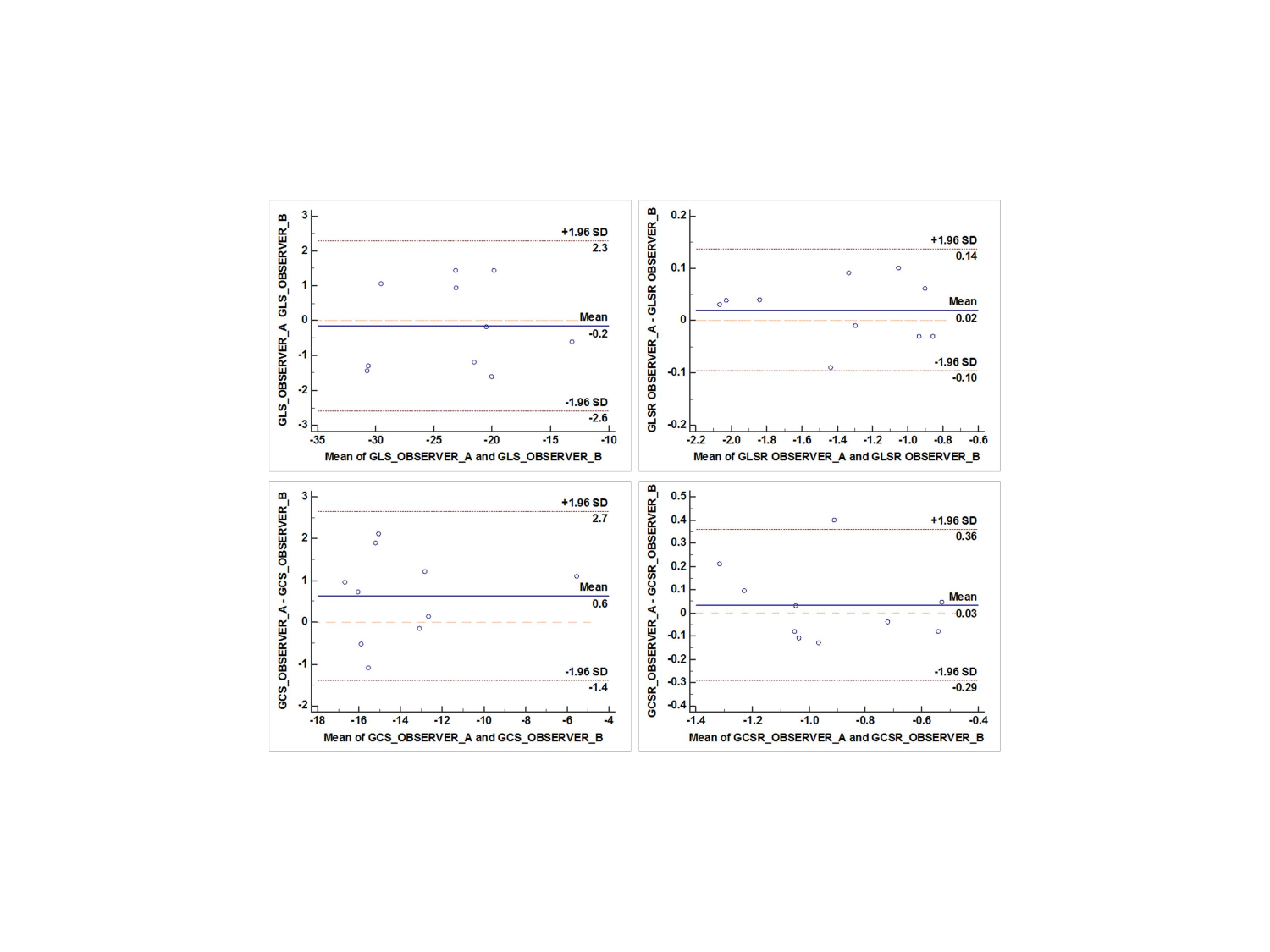

Supplement: Additional file 2: Figure S1. — Bland-Altman plots for inter-observer agreement. (PPTX 509 kb) [file 12968_2016_258_MOESM2_ESM.pptx]
