# Supplementary material for: Characterization and clinical significance of right ventricular mechanics in pulmonary hypertension evaluated with cardiovascular magnetic resonance feature tracking
Source: J Cardiovasc Magn Reson. 2016 Jun 16;18:39. doi: 10.1186/s12968-016-0258-x (PMC4910232; doi:10.1186/s12968-016-0258-x)
Supplement: Additional file 3: Table S2. — Correlation coefficients of right ventricular strain indices (absolute values) with CMR and RHC-derived parameters. (DOCX 82 kb) [file 12968_2016_258_MOESM3_ESM.docx]

**Supplemental Table 2. Correlation coefficients of right ventricular strain indices (absolute values) with CMR and RHC-derived parameters**

| Parameter | GLS | GLSR | GCS | GCSR |
| --- | --- | --- | --- | --- |
| RVEF | 0.72 (p<0.001) | 0.47 (p<0.001) | 0.72 (p<0.001) | 0.58 (p<0.001) |
| RVEDV index | -0.52 (p<0.001) | -0.42 (p<0.001) | -0.53 (p<0.001) | -0.45 (p<0.001) |
| RVESV index | -0.59 (p<0.001) | -0.44 (p<0.001) | -0.60 (p<0.001) | -0.48 (p<0.001) |
| Right atrial area | -0.45 (p<0.001) | -0.38 (p<0.001) | -0.30 (p =0.001) | -0.32 (p<0.001) |
| RV mass index | 0.46 (p<0.001) | 0.52 (p<0.001) | 0.40 (p<0.001) | 0.48 (p<0.001) |
| Mean PA pressure | -0.54 (p<0.001) | -0.42 (p<0.001) | -0.49 (p<0.001) | -0.51 (p<0.001) |
| PA oxygen saturation | 0.40 (p<0.001) | 0.33 (p<0.001) | 0.56 (p<0.001) | 0.32 (p=0.001) |
| Pulmonary vascular resistance index | -0.35 (p<0.001) | -0.42 (p<0.001) | -0.31 (p=0.001) | -0.34 (p<0.001) |
| Cardiac index | 0.19 (p=0.048) | 0.38 (p<0.001) | 0.12 (p=0.21) | 0.14 (p=0.14) |

GCS = global circumferential strain; GCSR = global circumferential strain rate; GLS = global longitudinal strain; GLSR = global longitudinal strain rate; PA = pulmonary artery; RVEDV = right ventricular end-diastolic volume; RVEF = right ventricular ejection fraction; RVESV = right ventricular end-systolic volume.
