# Supplementary material for: Characterization and clinical significance of right ventricular mechanics in pulmonary hypertension evaluated with cardiovascular magnetic resonance feature tracking
Source: J Cardiovasc Magn Reson. 2016 Jun 16;18:39. doi: 10.1186/s12968-016-0258-x (PMC4910232; doi:10.1186/s12968-016-0258-x)
Supplement: Additional file 4: Table S3. — Strain parameters in patients with and without late gadolinium enhancement. (DOCX 47 kb) [file 12968_2016_258_MOESM4_ESM.docx]

**Supplemental Table 3. Strain parameters in patients with and without late gadolinium enhancement**

| Strain parameter | Late gadolinium enhancement | | P |
| --- | --- | --- | --- |
|  | Positive | Negative |  |
| GLS | -13.61 ± 4.9 | -19.12 ± 6.5 | <0.001 |
| GCS | -10.06 ± 3.5 | -13.21 ± 5.2 | 0.002 |
| GLSR | -0.8 ± 0.2 | -1.2 ± 0.4 | <0.001 |
| GCSR* | -0.67 [-0.79 - (-0.54)] | -0.91[-1.1 - (-0.7)] | <0.001 |

Values are mean ± standard deviation or median [interquartile range]

GCS = global circumferential strain; GCSR = global circumferential strain rate; GLS = global longitudinal strain; GLSR = global longitudinal strain rate.
