# Supplementary material for: Characterization and clinical significance of right ventricular mechanics in pulmonary hypertension evaluated with cardiovascular magnetic resonance feature tracking
Source: J Cardiovasc Magn Reson. 2016 Jun 16;18:39. doi: 10.1186/s12968-016-0258-x (PMC4910232; doi:10.1186/s12968-016-0258-x)
Supplement: Additional file 5: Table S4. — Cox proportional adjusted hazard ratio for final multivariate alternative model of each RV strain parameter. (DOCX 45 kb) [file 12968_2016_258_MOESM5_ESM.docx]

**Supplemental Table 4. Cox proportional adjusted hazard ratio for final multivariate alternative model of each RV strain parameter**

| Models | Hazard ratio | 95% Confidence Interval | P |
| --- | --- | --- | --- |
| GLS  Cardiac index | 1.06  0.59 | 1 - 1.12  0.4 - 0.79 | **0.026**  **0.030** |
| RVEF  GLSR | 0.97  2.8 | 0.95 - 0.99  1.1 - 7.07 | **0.009**  **0.02** |
| RVEF* | 0.96 | 0.94 - 0.98 | **0.001** |
| GCSR  Cardiac index | 6.4  0.70 | 2.1 - 19.4  0.54 - 0.90 | **0.001**  **0.006** |

*Model including global circumferential strain, not shown.
