# Supplementary material for: Characterization and clinical significance of right ventricular mechanics in pulmonary hypertension evaluated with cardiovascular magnetic resonance feature tracking
Source: J Cardiovasc Magn Reson. 2016 Jun 16;18:39. doi: 10.1186/s12968-016-0258-x (PMC4910232; doi:10.1186/s12968-016-0258-x)
Supplement: Additional file 6: Table S5. — Cox proportional adjusted hazard ratio for final multivariate model including all RV strain parameters. (DOCX 39 kb) [file 12968_2016_258_MOESM6_ESM.docx]

**Supplemental Table 5.** Cox proportional adjusted hazard ratio for final multivariate model including all RV strain parameters

| Models | Hazard ratio | 95% Confidence Interval | P |
| --- | --- | --- | --- |
| GCSR  Cardiac index  Mean PA pressure  RV mass index | 4.3  0.6  0.97  1.02 | 1.2 - 15  0.45 - 0.80  0.95 - 0.99  1 - 1.04 | **0.02**  **0.01**  **0.02**  **0.005** |
|  |  |  |  |

GCSR = global circumferential strain rate
